# Survival of *Staphylococcus aureus* ST398 in the Human Nose after Artificial Inoculation

**DOI:** 10.1371/journal.pone.0048896

**Published:** 2012-11-14

**Authors:** Bibi C. G. C. Slingerland, Mehri Tavakol, Alex J. McCarthy, Jodi A. Lindsay, Susan V. Snijders, Jaap A. Wagenaar, Alex van Belkum, Margreet C. Vos, Henri A. Verbrugh, Willem J. B. van Wamel

**Affiliations:** 1 Department of Medical Microbiology and Infectious Diseases, Erasmus MC, Rotterdam, The Netherlands; 2 Division of Clinical Sciences, Centre for Infection, St George's University of London, London, United Kingdom; 3 Department of Infectious Diseases and Immunology, Faculty of Veterinary Medicine, Utrecht University, Utrecht, The Netherlands; 4 Central Veterinary Institute of Wageningen UR, Lelystad, The Netherlands; National Institutes of Health, United States of America

## Abstract

There is evidence that MRSA ST398 of animal origin is only capable of temporarily occupying the human nose, and it is therefore, often considered a poor human colonizer.

We inoculated 16 healthy human volunteers with a mixture of the human MSSA strain 1036 (ST931, CC8) and the bovine MSSA strain 5062 (ST398, CC398), 7 weeks after a treatment with mupirocin and chlorhexidine-containing soap. Bacterial survival was studied by follow-up cultures over 21 days. The human strain 1036 was eliminated faster (median 14 days; range 2–21 days) than the bovine strain 5062 (median 21 days; range 7–21 days) but this difference was not significant (*p* = 0.065). The bacterial loads were significantly higher for the bovine strain on day 7 and day 21. 4/14 volunteers (28.6%) showed elimination of both strains within 21 days. Of the 10 remaining volunteers, 5 showed no differences in bacterial counts between both strains, and in the other 5 the ST398 strain far outnumbered the human *S. aureus* strain. Within the 21 days of follow-up, neither human strain 1036 nor bovine strain 5062 appeared to acquire or lose any mobile genetic elements. In conclusion, *S. aureus* ST398 strain 5062 is capable of adequately competing for a niche with a human strain and survives in the human nose for at least 21 days.

## Introduction

*Staphylococcus aureus* (*S. aureus*) is a well known pathogen and is capable of colonizing the skin and mucosa of humans with the anterior nares being the most common carriage site [1]. Three human nasal carriage patterns can be distinguished: the persistent (30%), intermittent (40%) and non-carriage (30%) pattern [2]. This was recently reduced to two major phenotypes: persistent and non-carriage only [3]. Importantly, nasal carriage of *S. aureus* increases the risk for infection with this bacterial species [4]. The control of methicillin-resistant *S. aureus* (MRSA) reservoirs and infections is often problematic because these populations are resistant to almost al β-lactam antibiotics, the treatment of choice for Staphylococcal infections, and are often resistant to other commonly prescribed antibiotics [5]. Recently there has been a worldwide change in the epidemiology of MRSA. MRSA populations have been a problem in hospitals worldwide since the 1960s, but the emergence of new clones of MRSA has occurred in the community among individuals who lacked contact with healthcare [6]. In the US nearly all MRSA are associated with the community-associated (CA)-MRSA USA300 clone [7]. Nowadays in many European countries, Northern Americas, Australia and Asia there has also been an increased incidence of carriage of a livestock-associated (LA)-MRSA, especially in people with direct contact with livestock, such as farmers and veterinarians [8]. The majority of these LA-MRSA cases are caused by MRSA multi-locus sequence type (ST) 398, a lineage that can be detected by the fact that strains are Pulsed Field Gel Electrophoresis (PFGE) non-typeable, by restriction-modification (RM) testing and PCR testing [9], [10].

Currently ST398 MRSA/MSSA is reported in hospitals where it caused a broad spectrum of relatively mild infections including soft skin and tissue infections (SSTI) [11], [12], abscesses, urinary tract infections (UTI) and wound infections [13], [14]. In rare cases severe infections such as endocarditis [15] and bacteraemia [13] have been observed, although these occurred in older patients with underlying diseases. Often these cases were livestock-associated but occasionally infections occurred in people lacking contact with livestock.

The level of intensity and the duration of direct contact with livestock are important factors in proving positive for MRSA ST398. Prevalence of MRSA ST398 in farm-workers decreases substantially during holidays and in periods of less intense contact with livestock [16]. Van Cleef et al. showed that humans, who are temporarily in close contact with livestock, easily acquire MRSA ST398 but also shed the strain in less than 24 hours [17]. Whilst, in the hospital environment, Wassenberg et al. showed that nosocomial transmission of ST398 is 72% less likely to occur compared to non-ST398 strains [18]. It remains unclear whether these low rates of transmission and persistence are pathogen or patient-related.

*S. aureus* adapt to different host environments by acquiring mobile gene elements (MGEs) carrying genes encoding host-specific immune evasion strategies. The best characterised human-specific factor is the φ3 bacteriophage that carries the immune evasion cluster (IEC) genes *chp*, *sak* and *scn* [19]. This bacteriophage is commonly found in human *S. aureus* but is rare amongst animal *S. aureus* [20], [21]. Recently it was shown that many ST398 isolates of human origin do not carry the φ3 bacteriophage or any of the IEC genes suggesting that ST398 can survive and even cause infection in the human host without acquisition of the φ3 bacteriophage [22].

At the moment data concerning the intrinsic capacity of ST398 to colonize the human nose are lacking. In this study, we undertook an artificial human inoculation experiment with a mixed inoculum of a bovine MSSA ST398 (CC398) strain and human MSSA ST931 (CC8) strain, 7 weeks after a treatment with mupirocin and chlorhexidine-containing soap, and determined their ability to survive in the anterior nares. In addition, we used microarray analysis to compare the genomes of parental strains and strains that survived in the nose.

## Materials and Methods

### Study population

Twenty-two healthy volunteers were included in this study (seven males and fifteen females, median age of 27 years, range 19–57 years). An infectious disease physician was on call for the entire study period and all volunteers provided their written informed consent. The study protocol was approved by the local Medical Ethical Committee of the Erasmus University Medical Centre Rotterdam, The Netherlands (MEC-2011-131).

### *S. aureus* strains

The human *S. aureus* strains (502A, 274, 1036, P1, P2 and I) were all used in earlier inoculation experiments [3], [23]. The bovine MSSA ST398 strains used in this study were obtained in 2008 from healthy calves in The Netherlands as part of a MRSA prevalence study [24]. Of the MSSA ST398 strains we also obtained MRSA counterparts, which were isolated from the same calf. For determination of the genetic background MLST analyses [25] and *spa*-typing were performed [26]. The *agr* locus was amplified to determine the *agr*-type (1–4) [27]. The detection of *sea* – *seu*, *tst* [28], *eta*, *etb* and *lukS/lukF* [29] was performed by PCR. PCR analysis with ST398-specific primer set A07 [10] was performed. The VITEK (bioMérieux, Marcy l'Etoile, France) was used to determine the antibiotic susceptibility of the strains. For a second opinion, the strains were sent to the National Institute for Public Health and the Environment (RIVM, Bilthoven, The Netherlands), and were analyzed for toxin production. Bacterial growth rates of the strains were determined in Brain Heart Infusion (BHI) and Tryptic Soy Broth (TSB). Bacteria were grown for 7 hours at 37°C.

### Artificial inoculation protocol

The artificial inoculation protocol was as described previously [3], [20], [30]. In brief, before inoculation the carriage state for *S. aureus* was determined by taking two nasal swabs with an interval of one week. We defined carriers as persons with two consecutive nasal swabs culture-positive for *S. aureus*. A non-carrier had one or no positive nasal cultures. Blood was drawn in week 1 to determine C-reactive protein (CRP) levels (mg/L) and the leukocyte number (*10E9/L). After determining their carriage state, all volunteers were instructed to use mupirocin nasal ointment (2%; GlaxoSmithKline, Waltham, MA, USA) twice daily and chlorhexidine-containing soap once daily for five days as eradication treatment for *S. aureus*. The volunteers received hygienic advice and medical check-ups. These medical check-ups included questions about signs of infection and when indicated physical examination. Volunteers remaining positive for *S. aureus* after eradication treatment were excluded from the inoculation phase. Therefore, the anterior nares were again cultured six weeks after the treatment to determine if the treatment had been successful. Artificial inoculation was performed in successfully decolonized volunteers one week later by applying 10*7 CFU per strain in the left and right nostril using a 1∶1 mixture of human strain 1036 and bovine strain 5062. In the follow-up period the anterior nares were cultured on day 1, 2, 4, 7, 10, 14 and 21 after inoculation. At the end of the study, pharyngeal and perineal swabs were cultured as well. CRP and the leukocyte number were again determined on day 21. Eradication treatment was given to volunteers still carrying the inoculated *S. aureus* strain(s) at the end of follow-up.

### Nasal swab cultures

The nose was sampled by revolving a single swab around both the left and right anterior nares four times. Swabs were submerged in Stuart's transport medium and vortexed (15 s). Swab eluates were cultured quantitatively at 37°C via plating of serial dilutions of the eluates on phenol red mannitol salt agar plates (PHMA) (2 days). The eluted swabs were submerged and incubated in phenol red mannitol salt broth (PHMB) for 7 days at 37°C. The PHMA culture plates were incubated at room temperature for 5 further days. Both strain 1036 and 5062 are morphologically distinguishable by colony colour which was used to determine the total density of each strain per swab. Furthermore, strain 5062 ferments lactose while strain 1036 does not. Therefore, twenty-five colonies of each morphotype were selected by colour and placed on lactose agar plates for verification. A latex agglutination test (Slidex Staph Plus, bioMérieux, Marcy-l'Etoile, France) was performed for suspected colonies (yellow colour and/or haemolytic zone). For final identification, ten out of the twenty-five isolates of each morphotype were analyzed by PCR-analysis of the *spa*-gene.

### Microarray analysis

Microarray experiments were performed using a 62-strain *S. aureus* microarray (SAM-62), as previously described (McCarthy et al. 2011). SAM-62 contains 29,739 60-mer oligo probes representing 6,520 genes, and an additional 579 gene variants, from the first 62 sequenced *S. aureus* genomes and from 153 sequenced plasmid genomes. The array design is available in BμG@Sbase (Accesion No. A-BUGS-38; http://bugs.sgul.ac.uk/A-BUGS-38) and ArrayExpress (Accession No. A-BUGS-38). All data analysis was performed in GeneSpring GX v11.01 (Agilent Technologies). Fully annotated microarray data have been deposited in BμG@Sbase (accession number E-BUGS-131; http://bugs.sgul.ac.uk/E-BUGS-131) and also ArrayExpress (accession number E-BUGS-131).

### Statistical analysis

Statistical analyses were performed with SPSS, version 17.0 (SPSS Inc., Chicago, IL, USA). The primary outcome after artificial inoculation was the survival time of *S. aureus* in the nose. We defined survival time as the time in days until the final positive nasal culture for each of the inoculated *S. aureus* strains. A Kaplan-Meier survival analysis (log-rank test) was used to compare survival between the human and bovine strain. The Mann-Whitney *U*-test was used to compare median number of CFUs. *P*<0.05 was considered statistically significant.

## Results

### *S. aureus* strains

All *S. aureus* strains were extensively analysed for genetic background, toxin gene content, *agr*-type and antibiotic resistance profile. For obvious ethical reasons, MSSA deficient in toxin genes, sensitive to as many antibiotics possible, sharing the same *agr*-type and growth characteristics were selected for the inoculation experiment. Therefore, out of the initially 6 human isolates, we selected strain 1036 which belonged to ST931 (CC8). The strain was susceptible to all common antimicrobials (e.g. mupirocin, flucloxacillin and vancomycin), had *agr*-type 1 and lacked superantigens (*sea – seu, tst*), enterotoxins (*eta, etb*) and *lukS/lukF*.

The selected bovine strain was 5062 and belonged to ST398 (CC398). This strain was resistant to tetracycline and trimethoprim, but was susceptible to other common antimicrobials and was devoid of the staphylococcal toxins mentioned above. This strain had *agr*-type 1 and *spa*-type t034. PCR analysis with primer set A07 confirmed that strain 5062 was of the ST398 lineage. The MRSA ST398 counterpart that was isolated from the same calf shared the same *spa*-type, PFGE-type and other characteristics (e.g. gene content, antibiotic susceptibility) except for the presence of the *mecA*-gene. The RIVM confirmed that both the human and bovine strain was not producing the staphylococcal toxins mentioned above. Growth kinetics in Brain-Heart Infusion (BHI) were comparable for strain 1036 and 5062 (data not shown).

### Artificial nasal inoculation with human strain 1036 and bovine strain 5062

A total of 22 volunteers were included in the study and their carriage state was determined. Six volunteers (27%) were classified as carriers and sixteen volunteers (73%) were classified as noncarriers. All volunteers used the eradication treatment as described and six weeks after the treatment five carriers were positive for *S. aureus* and they were therefore excluded from further intervention and follow-up. One participant was excluded because of private reasons. The remaining sixteen volunteers (1 carrier; 15 non-carriers) were inoculated with a mixture of the two strains seven weeks after the treatment. Two volunteers were excluded from further analyses because of the use of antibiotics during the study period. One started antibiotics for UTI on the day of inoculation; the other participant developed an intranasal furuncle which was treated. All volunteers adhered to the study protocol.

**Figure 1:**
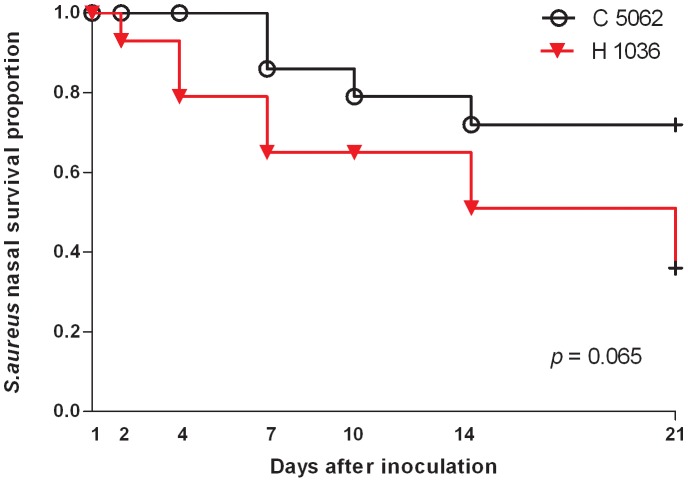
Survival of the inoculated strains. Kaplan-Meier survival curves showing the proportion of volunteers who are *S. aureus* culture-positive after the artificial inoculation with human strain 1036 and bovine strain 5062.

*S. aureus* survival was determined by quantitative cultures of 7 consecutive nasal swabs (day 1, 2, 4, 7, 10, 14, 21) in a period of 21 days. The Kaplan-Meier curves in Figure 1 show the proportion of positive cultures during follow-up. Overall the human strain 1036 was eliminated at a faster pace (median 14 days; range 2–21 days) than the bovine strain 5062 (median 21 days; range 7–21 days) but this difference was not significant (*p* = 0.065).

**Figure 2:**
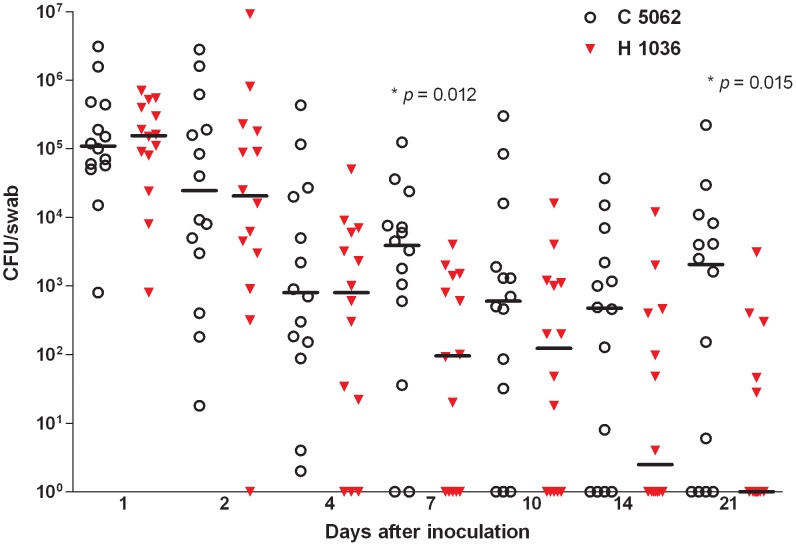
Bacterial loads in the nares of volunteers after inoculation. Each dot represents the number of CFUs per swab at day 1, 2, 4, 7, 10, 14 and 21 after the inoculation with the mixture of *S. aureus* strain 1036 of human origin and strain 5062 of bovine origin. The horizontal bars represent the median number of CFUs at indicated sampling times.

The densities of the two strains in the anterior nares rapidly decreased during the first days after inoculation. When the differences in bacterial counts between the human and bovine strain were studied on day 7 and 21, significantly higher densities were found for the bovine strain in comparison to the human strain (respectively *p* = 0.012 and *p* = 0.015) (Fig. 2).

**Figure 3:**
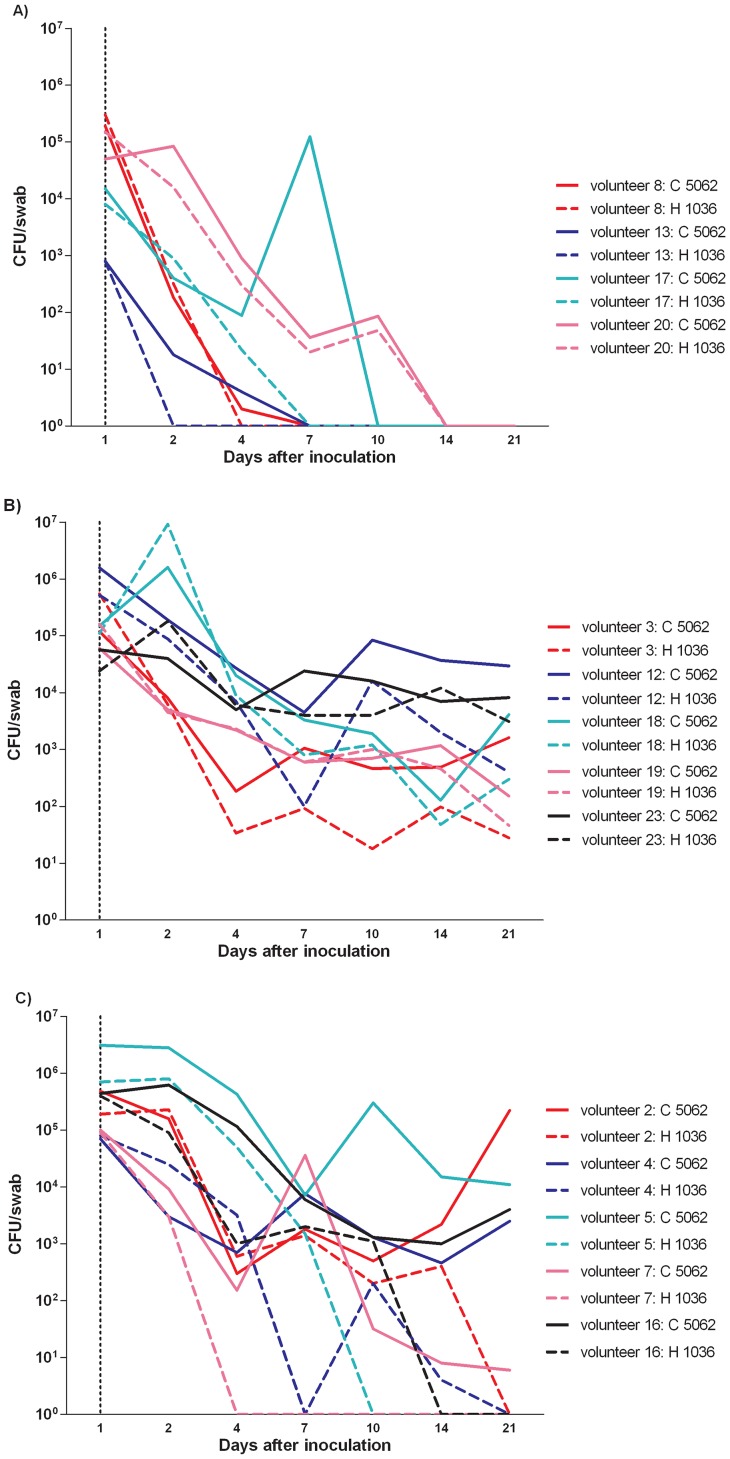
Three elimination patterns. Each line represents the number of CFUs of each volunteer at day 1, 2, 4, 7, 10, 14 and 21 after the inoculation with the mixture of *S. aureus* strain 1036 of human origin and strain 5062 of bovine origin. Fig. 3A shows the data of those volunteers who eliminated both strains within 21 days. Fig. 3B shows those volunteers in whom both strains showed similar elimination rates and Fig. 3C those where the bovine strain survived more successfully than the human strain.

Two patterns of elimination of the inoculated strains were observed. One group of volunteers (28.6%, *n* = 4/14; 4 non-carriers) showed elimination of both strains within 21 days (Fig. 3A), while in the remaining 10 volunteers (71.4%; 1 carrier, 9 non-carriers) *S. aureus* was culture-positive up to day 21 until the end of the study. Interestingly, this last group of 10 volunteers could be further sub-divided. In 5 volunteers (1 carrier, 4 non-carriers) no differences in bacterial counts between both strains were observed during the 21 days (Fig. 3B). In contrast, in the remaining 5 volunteers (all non-carriers) strain 5062 far outnumbered strain 1036 (Fig. 3C).

An increase in bacterial load of the bovine strain at the end of the follow-up period could indicate adaptation to the host or waning of the host immune response. In order to study if acquisition of MGEs by (one of) the inoculated strains could have occurred, microarray analysis was performed on both parent strains as well as on every last positive culture isolate for strain 1036 and 5062 from the 10 volunteers who showed no elimination of both strains within 21 days. The parent human strain 1036 carried a *S. aureus* pathogenicity island (SaPI)2, plasmids with *rep* genes *rep*_5_, *rep*_21_, *rep*_23_ and *rep*_30_, and genes *cadDX* and *qacA* conferring resistance to cadmium and antiseptics (Figure S1). This strain did not carry the φ3 bacteriophage or any of the IEC genes. The parent bovine strain 5062 carried the φ6 bacteriophage and the *tetM* and *dfrG* genes encoding resistance to tetracycline and trimethoprim.

After 21 days of follow-up, the bovine 5062 strains did not acquire any MGE from the human strains in any of the 10 volunteers. In addition, no bovine strain lost an MGE that was present in the parental strain, although microheterogeneity was detected in some of these bovine strains. Likewise, the human 1036 strains did not acquire or lose any MGEs. In these human strains microheterogeneity was also seen, but this was not due to acquisition of MGEs. We note that isolate H1036 from volunteer 23 has the φ6 integrase gene, but as it does not possess any other bacteriophage genes we are confident this is a false positive.

At the end of the study, all volunteers were in healthy condition. Laboratory values indicated no signs of infection. In five volunteers, all nasal swabs and two pharyngeal swabs were still positive for the inoculated bovine strain 5062. In five other volunteers, all nasal swabs and four pharyngeal swabs were positive for both of the inoculated strains. Eradication treatment was given to all these volunteers. Nasal and pharyngeal swabs following eradication treatment were all negative.

## Discussion

We demonstrate in an artificial human nasal inoculation model, that *S. aureus* ST398 of bovine origin is capable of surviving in the nose in 10 healthy volunteers for at least 21 days when inoculated 7 weeks after an eradication treatment with mupirocin and chlorhexidine-containing soap. We found no evidence that survival of ST398 in the human host was due to the acquisition of MGEs. There is evidence that MRSA ST398 of animal origin is only capable of temporarily occupying the human nose. It is, therefore, often considered as a poor human colonizer [17], [31]. Our study shows this loss of colonization in livestock workers is not due to an intrinsic inability of ST398 to survive in the human nose.

Van Cleef et al. showed that MRSA ST398 can easily be acquired but is also lost within 24 hours by those who are temporarily in close contact with livestock [17]. An explanation for the discrepancy between our data and that of van Cleef et al. could be the inoculum size and or immunological effect. In our inoculation experiment we used an inoculum of 10*7 bacteria per strain per nostril, but currently it is not known what the level of bacterial exposure is during an average day of farming. It could very well be that this is a much lower number of bacteria than the inoculum we used. Another difference between our study and exposure to ST398 on farms is that we pretreated all our volunteers with mupirocin, an intervention that may eradicate other elements of the nasal microflora, coagulase-negative staphylococci in particular, that play a role in the resistance of the nose against *S. aureus* colonization [32].

In the first days after inoculation we observed a rapid decrease in bacterial load of both strains in the nares of all volunteers, resulting in the elimination of both strains within 21 days in four volunteers. Interestingly, in the remaining 10 volunteers ST398 could still be detected after 21 days. In half of this latter group we observed, after a decrease in bacterial loads of both strains, that the loads stabilized after 21 days. In the remaining five individuals, cell counts for strain ST398 increased at the end of follow-up where in most of these cases the human strain was eliminated. Our data clearly indicate that in 28.6% of the volunteers *S. aureus* is rapidly eradicated even when exposed to significant numbers of bacteria. Yet, 71.4% of the volunteers were not able to eradicate either or one of the inoculated *S. aureus* strains.

We found no evidence that persistence of ST398 in the human host was due to the acquisition of MGEs. This suggests that animal ST398 is able to survive for several weeks in the human nares without gaining or losing MGEs. This agrees with a previous study that showed that human-specific φ3 bacteriophage and the IEC genes encoding *chp, sak* and *scn* are absent in the majority of ST398 isolates from humans [22]. How does ST398 colonize different host species? *S. aureus* encode multiple surface proteins that interact with host ligands, and many of these proteins often have overlapping functions and can function in multiple hosts [33].

In conclusion, MSSA strain 5062 of bovine origin (ST398, *spa*-type t034) is capable of surviving in the human nose for at least 21 days where it appears to successfully compete with human strain 1036.

## Supporting Information

Figure S1 **SAM-62 microarray analysis of parent strains and colonizing isolates in 10 human volunteers** Isolates are represented by vertical lines and information about the origin of each isolate is given at the top of the figure. Group 1 are the volunteers in whom no difference in bacterial load between both strains was observed (Fig. 3B) and Group 2 are those who did show a difference in bacterial load between both strains at the end of follow-up (Fig. 3C). Horizontal lines represent 57 different 60-mer oligo probes specific to 5 *hsdS* variants, 4 bacteriophage genes, 1 SaPI gene, 4 plasmid *rep* families, and 5 different antimicrobial, biocide and heavy metal resistance genes. The colour depicts if the gene is present or absent in the respective isolate; red or yellow = present, blue or black = absent. After 21 days of follow-up, neither human strains 1036, nor bovine strains 5062 acquired or lost any MGEs.
(TIF)
