# Supplementary figures and images for: Survival of Staphylococcus aureus ST398 in the Human Nose after Artificial Inoculation
Source: PLoS One. 2012 Nov 14;7(11):e48896. doi: 10.1371/journal.pone.0048896 (PMC3498341; doi:10.1371/journal.pone.0048896)

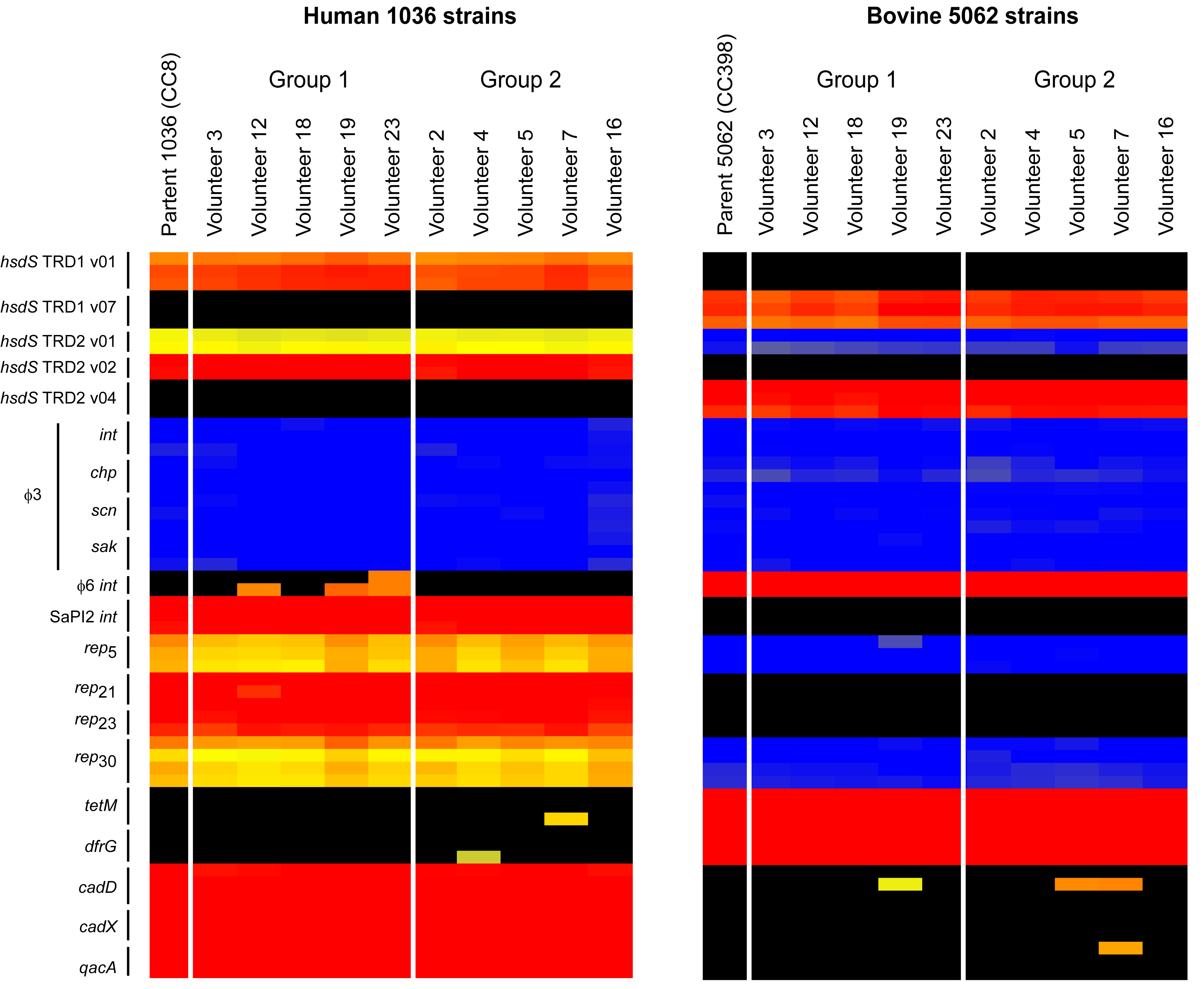

Supplement: Figure S1 — SAM-62 microarray analysis of parent strains and colonizing isolates in 10 human volunteers Isolates are represented by vertical lines and information about the origin of each isolate is given at the top of the figure. Group 1 are the volunteers in whom no difference in bacterial load between both strains was observed (Fig. 3B) and Group 2 are those who did show a difference in bacterial load between both strains at the end of follow-up (Fig. 3C). Horizontal lines represent 57 different 60-mer oligo probes specific to 5 hsdS variants, 4 bacteriophage genes, 1 SaPI gene, 4 plasmid rep families, and 5 different antimicrobial, biocide and heavy metal resistance genes. The colour depicts if the gene is present or absent in the respective isolate; red or yellow = present, blue or black = absent. After 21 days of follow-up, neither human strains 1036, nor bovine strains 5062 acquired or lost any MGEs. (TIF) [file pone.0048896.s001.tif]
